# *MS4A2*-rs573790 Is Associated With Aspirin-Exacerbated Respiratory Disease: Replicative Study Using a Candidate Gene Strategy

**DOI:** 10.3389/fgene.2018.00363

**Published:** 2018-09-11

**Authors:** Gandhi F. Pavón-Romero, Gloria Pérez-Rubio, Fernando Ramírez-Jiménez, Enrique Ambrocio-Ortiz, Elisé Bañuelos-Ortiz, Norma Alvarado-Franco, Karen E. Xochipa-Ruiz, Elizabeth Hernández-Juárez, Beatriz A. Flores-García, Ángel E. Camarena, Luis M. Terán, Ramcés Falfán-Valencia

**Affiliations:** ^1^Department of Immunogenetics and Allergy, Instituto Nacional Enfermedades Respiratorias Ismael Cosío Villegas, Mexico City, Mexico; ^2^HLA Laboratory, Instituto Nacional Enfermedades Respiratorias Ismael Cosío Villegas, Mexico City, Mexico; ^3^Biomedicine in the Post-Genomic Era, Mexico City, Mexico

**Keywords:** AERD, *MS4A2*, genetic association, SNP, replicative study, aspirin intolerant asthma

## Abstract

Aspirin exacerbated respiratory disease (AERD) is a set of diseases of the unified airway, and its physiopathology is related to disruption of the metabolism of arachidonic acid (AA). Genetic association studies in AERD had explored single nucleotide polymorphism (SNPs) in several genes related to many mechanisms (AA metabolism, inflammation, drug metabolism, etc.) but most lack validation stages in second populations. Our aim is to evaluated whether contribution to susceptibility of SNPs reported in other populations are associated with AERD in Mexican Mestizo patients. We developed a replicative study in two stages. In the first, 381 SNPs selected by fine mapping of associated genes, (previously reported in the literature), were integrated into a microarray and tested in three groups (AERD, asthma and healthy controls -HC-) using the GoldenGate array. Results associated to risk based on genetic models [comparing: AERD vs. HC (comparison 1, C1), AERD vs. asthma (C2), and asthma vs. HC (C3)] were validated in the second stage in other population groups using qPCR. In the first stage, we identified 11 SNPs associated with risk in C1.The top SNPs were *ACE-*rs4309C (*p* = 0.0001) and *MS4A2-*rs573790C (*p* = 0.0002). In C2, we detected 14 SNPs, including *ACE-*rs4309C (*p* = 0.0001). In C3, we found *MS4A2-*rs573790C (*p* = 0.001). Using genetic models, C1 *MS4A2-*rs57370 CC (*p* = 0.001), and *ACE-*rs4309 CC (*p* = 0.002) had associations. In C2 *ACE-*rs4309 CC (*p* = 0.0001) and C3 *MS4A2-*rs573790 CC (*p* = 0.001) were also associate with risk. In the second stage, only *MS4A2-*rs573790 CC had significance in C1 and C3 (*p* = 0.008 and *p* = 0.03). We concluded that rs573790 in the *MS4A2* gene is the only SNP that supports an association with AERD in Mexican Mestizo patients in both stages of the study.

## Introduction

Aspirin exacerbated respiratory disease (AERD) is an illness characterized by chronic rhinosinusitis with nasal polyps, asthma and hypersensitivity to non-steroidal anti-inflammatory drugs (NSAIDs) such as acetylsalicylic acid (ASA) (Lee and Stevenson, 2010). Its prevalence depends on the reference consulted, ranging from 7% using specific questionnaires to 21% when provocation tests are used (Jenkins et al., 2004; Rajan et al., 2015). The physiopathology mechanism is not yet understood. The principal hypotheses is the disruption of acid arachidonic (AA) metabolism by the pharmacologic action of ASA or NSAID, the blockage of cyclooxygenase (COX) type 2 from the COX pathway to the lipoxygenase pathway with the subsequent increase in the synthesis of leukotrienes (LTC4, LTD4 and LTE4), immunological agents responsible for histopathologic changes, and the severity of the characterized symptoms of AERD (Laidlaw and Boyce, 2013; Thompson et al., 2016). Recently, new mechanisms have been integrated, such as epithelial damage mediated by thymic stromal lymphopoietin with activation of the innate type 2 immune system (Buchheit et al., 2016; Laidlaw and Boyce, 2016) and the involvement of the IL1β-IL1 axis in macrophages and eosinophils, increasing pro-inflammatory effects (Machado-Carvalho et al., 2016). AERD treatment consists of avoiding NSAIDs and controlling the pathologies that are integrated with it, together with nasal and inhaled steroids plus an antagonist of leukotrienes receptors (Montelukast), inclusive surgery for nasal polyps and desensitization with ASA for specific conditions (i.e., asthma control, recurrent polyps, and ASA for cardiovascular prevention) (Fokkens et al., 2012; GINA, 2016).

At first, genetic association studies in AERD were performed in genes related to the metabolism of AA, first with direct association with a risk allele, and then with genetic models (co-dominant, recessive and dominant) and clinical markers (methacholine or ASA hyperbronchial reactivity and eosinophilia). Additionally, this methodological strategy was used in studies with other types of genes implicated in inflammation, tissue damage, intracellular signaling, drug metabolism and antigen presentation. These investigations have been primarily performed in Korean population (Kim et al., 2013; Pavón-Romero et al., 2017). Recently, new methods evaluated the whole genome with techniques such as GWAS (genome-wide association study) to identify new candidate genes and/or SNP (single nucleotide polymorphisms) for screening susceptible populations to this entity, or predictive markers with therapeutic efficacy (Park et al., 2013; Kim et al., 2014). It is unknown whether this genetic background is applied to other populations, such as Latinos, specifically the Mexican Mestizo.

## Materials and methods

### Design study

**Figure 1:**
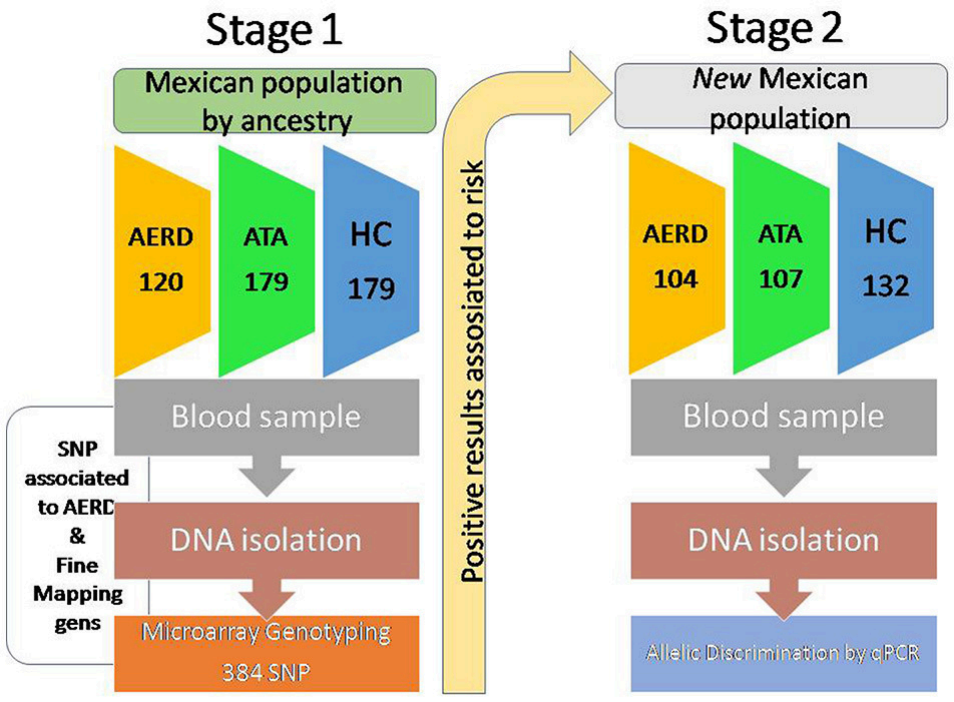
Design of study.

We developed a replicative study in two stages. In the first stage, we evaluated SNPs selected by fine mapping genes associated positively with AERD in three groups (AERD, asthma and healthy control -HC) using the GoldenGate array (Illumina, Inc., San Diego CA, USA), and only the positive results were validated in the second stage in another population of subjects with the same inclusion criteria using real-time PCR (Figure 1).

### Subjects

The first stage of the study included 478 subjects in three groups: 120 patients with AERD, 179 with asthma and 179 healthy controls, enrolled in asthma screening campaigns at the Immunogenetic & Allergy Department of the Instituto Nacional de Enfermedades Respiratorias Ismael Cosio Villegas (INER) at Mexico City. All subjects were Mexican-mestizo, defined as being born in Mexico and with Mexican ancestry (at least 2 previous generations) and not being from any particular ethnic group. AERD was defined as the presence of nasal polyps or antecedent of polyp surgery with intolerance to NSAID or ASA (nasal challenge with lysin-aspirin or antecedent of two severe reactions after the intake of NSAID or ASA, i.e., bronchospasm, documented in medical records) plus asthma. Asthma was established as persistent typical symptoms: shortness of breath, wheezing, chest tightness, and cough; plus ≥12% or 200 ml increase of forced expiratory volume in the first second (FEV_1_); and post-bronchodilatator spirometry (Mater Screen, Jaegger-Germany). If they had no clinical symptoms or positive tests, the subjects were classified as HC. Allergy sensitization was evaluated with a skin prick test, comprising 40 allergens (Alk-abello; Massachusetts, USA), and the levels of total IgE (Architect i2000, Roche, Germany) and eosinophils count in the blood were measured by hematic cytometry (Beckman Coulter LH750, USA).

The second stage included 104 patients with AERD, 105 asthma patients, and 132 HC unrelated to the first stage with the same classification criteria.

All subjects are residents from urban metropolitan area of Mexico City.

### DNA isolation

All subjects donated eight milliliters of peripheral blood by venipuncture collected in a tube with EDTA as anticoagulant. Subsequent DNA extraction was performed using a BDtract DNA Isolation Kit (Maxim Biotech; San Francisco, California, USA). The DNA was quantified by ultraviolet absorption at a 260-nm wavelength using a Nanodrop instrument (Thermo Scientific; DE, USA). All samples were adjusted to 50 ng/μl for subsequent genotyping.

### SNP selection and goldengate genotyping

**Figure 2:**
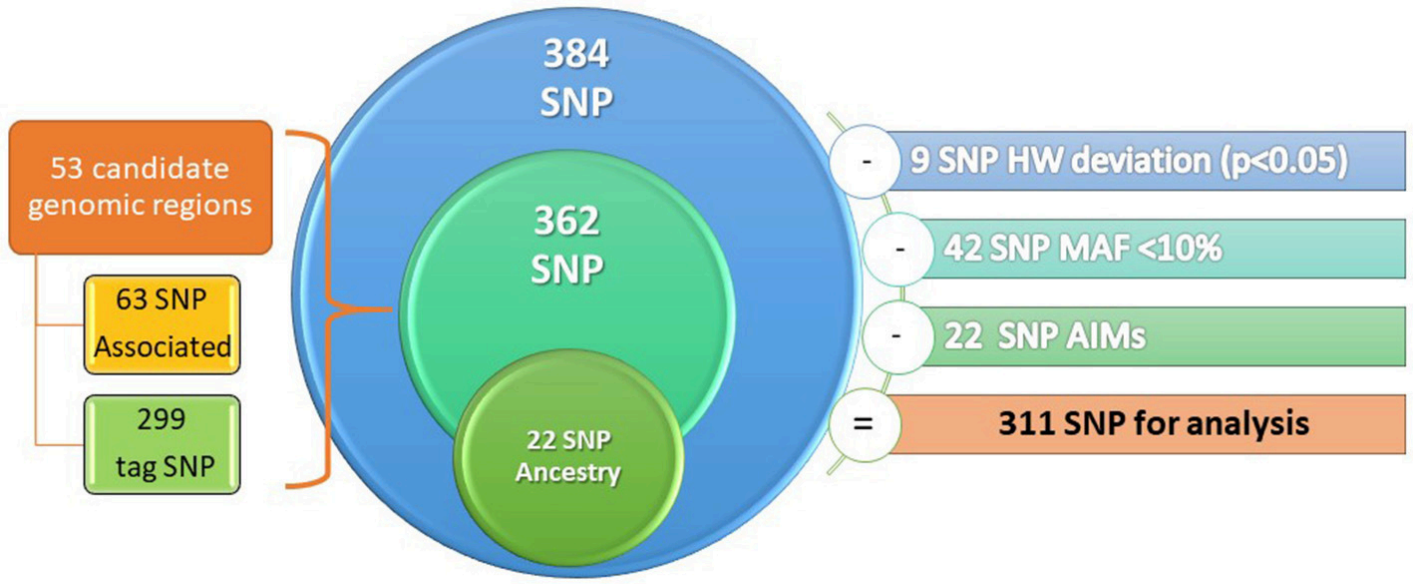
Design of genetic microarray.

An Illumina 384 SNP custom GoldenGate array was employed (Illumina Inc.; San Diego, CA, USA). The SNPs were selected according to a search of the US National Library of Medicine with the keywords *SNP* and *AERD, ASA hypersensitivity* and *SNP* between 1997 and 2014.The array included 384 SNPs from 53 candidate genomic regions spanning over 19 chromosomes, of which 63 SNPs were associated with AERD, 299 were tag SNPs, and 22 SNPs were ancestry informative markers (AIMs), which must to have a difference with respect to Caucasian (CEU) group of 30% to be considered as AIMs. The selection criteria of the SNPs were based on the minor allele frequency (MAF) >10% in Mexican mestizo population (data obtained from Mexican genome diversity project, MGDP) and with Hardy-Weinberg equilibrium *p* > 0.05 (Figure 2).

### Genotyping and quality control

Genotyping was conducted using the protocol designed by Illumina for the GoldenGate platform (Illumina, Inc.; San Diego, CA, USA) using a Tecan robotic automatic liquid dispenser (Tecan, Trading AG, Switzerland), which operates under the Illumina protocol. The microarrays were read on the BeadArray Reader scanner (Illumina, Inc.; San Diego, CA, USA). Genotype acquisition and generation of documentation (ped and map files) were conducted using the GenomeStudio 2011 v1.0 software (Illumina, Inc., San Diego CA, USA). Subjects who did not comply with the call rate criteria (>95%) were excluded.

### Taqman allelic discrimination

Genotyping in the second stage was performed using TaqMan allelic discrimination real-time PCR with predesigned probes in a7300 Real-Time PCR System (Applied Biosystems, Foster City CA, USA). This stage was performed using independent samples with the same characteristics as those samples used in the first stage. Genotype assignment was performed based on the allelic discrimination and confirmed by absolute quantitation. In addition, three non-template controls (contamination controls) were included for each genotyping plate, and 1% of the samples included in the study were genotyped in duplicate for control allele assignment. Data interpretation was conducted using the Sequence Detection Software (SDS v. 1.4, Applied Biosystems). VIC and FAM fluorophores were used for alleles A and B, respectively.

### *In silico* analysis

After validating the results in the second stage, we explored the theoretical role of the main SNPs in different biochemical processes, such as alterations in splicing using splice-site analysis with NetGene2 (http://www.cbs.dtu.dk/services/NetGene2). This program can be used to assess the presence of new binding sites for transcription factors and/or the creation or disruption of alternative splicing sites in the gene. To predict potential microRNAs (miRNAs), including the associated SNP and their potential target genes, the miRDB program (Wong and Wang, 2015) was used (http://www.mirbase.org/).

### Statistical analysis

In both stages, we analyzed clinical quantitative variables with non-parametric statistics, using SPSS software v.21 (SPSS software, IBM, New York, USA), and frequency analysis was performed with Epi-info software v.7.0. Generation of the fixation index (Fst) was performed using EIGENSOFT v4.2 software (Shringarpure and Xing, 2014). For the genetic association study, the software PLINK v1.07 (Purcell et al., 2007) was used, a logistic regression model (1° of freedom) was created that included co-variables such as age, sex; only, the genetic analysis of minor allele frequency was performed with PLINK software, subsequently, reanalyzing according to genetic models, the co-dominant and recessive models, were performed using the Epidat v. 3.1 software and Epi-info v. 7.2 software, respectively. These last two methods were applied for analysis in the second stage. In all analyses, we considered significance at a *p* < 0.05.

### Ethics

This study was reviewed and approved by the Bioethics and Science Committee in Research, with protocol number B14-12, and the Institutional Review Board at the Instituto Nacional de Enfermedades Respiratorias Ismael Cosio Villegas (INER). The participants were invited to join the study and were informed about the objective. They then signed an informed consent letter and were provided with an assurance-of-personal-data document. Each participant was assigned an alphanumeric key with the purpose of assuring confidentiality.

## Results

### Stage 1

#### Demographic and clinical data

In both stages, we conducted three comparisons: AERD vs. HC, AERD vs. asthma, and asthma vs. HC. In the first stage, we enrolled 478 subjects. The control group was younger than the patients (AERD or asthma, *p* < 0.01), and the female gender prevailed in the three groups (~60%). Eosinophil cell counts were higher in the AERD group than asthma and HC (*p* < 0.001). Serum total IgE had higher values in asthma than AERD patients and controls, positive allergy sensitivity was very similar in the three groups. A reversibility test at enrollment was positive in the asthma group, but not in the AERD, and it was negative in controls. Total nasal flow decreases after nasal lysin-aspirin challenge occurred only in the AERD group compared with the asthma and healthy control groups (*p* < 0.0001) (Table 1).

**Table 1:** Demographic and clinical data.

**Variable**	**First stage**						**Second stage**					
	**AERD**	**HC**	**A**	**AERD vs. HC**	**AERD vs. A**	**A vs. HC**	**AERD**	**HC**	**A**	**AERD vs. HC**	**AERD vs. A**	**A vs. HC**
*n*	120	179	179				104	132	107			
Age	43 (34–50)	27 (22–35)	39 (27–52)	^§^	NS	^§^	42 (33–53)	34 (26–43)	36 (27–46)	^§^	^¥^	NS
Female %	64.5	59.7	70.1	NS	NS	^¥^	70	73	74	^¥^	NS	^¥^
Eosinophils cell/mm^3^	400 (300–700)	100 (61–191)	170 (40–492)	^§^	^§^	^¥^	400 (215–700)	136 (84–219)	300 (200–428)	^§^	^¥^	^§^
IgE UI/L	125 (62–235)	88 (38–135)	229 (118–297)	^¥^	^¥^	^§^	107 (42–254)	63 (18–107)	266 (131–501)	^§^	^§^	^§^
Positive SPT %	46	52	55	NS	NS	NS	48	44	81	^¥^	^¥^	NS
FEV_1_ %^ϕ^	7 (2–13)	5 (0–5)	15 (8–21)	^§^	^¥^	^¥^	10 (5–13)	3 (0–5)	12 (6–16)	^¥^	^¥^	NS
NTF %^ϕϕ^	54 (48–59)	3.6 (−5 to 5)	10 (3–10)	^§^	^§^	^¥^	CA	–	–	–	–	–

AERD, Aspirin-Exacerbated Respiratory Disease; A, Asthma; CA, Clinical antecedent of acute asthma exacerbation before intake of aspirin; HC, Healthy Control; NTF, Nasal Total Flow (

ϕϕ percentage of change in lysin-aspirin challenge). SPT, Skin Prick Test; FEV_1_, Forced Expiratory Volume in the first second (

ϕ *percentage of change in reversibility test). All results of quantitative variables are in medians and interquartile range. Does not meet the criterion of positivity (FNT>40%)*.

¥ *p ≤ 0.05*.

§ *p ≤ 0.0001*.

#### Ancestry

**Figure 3:**
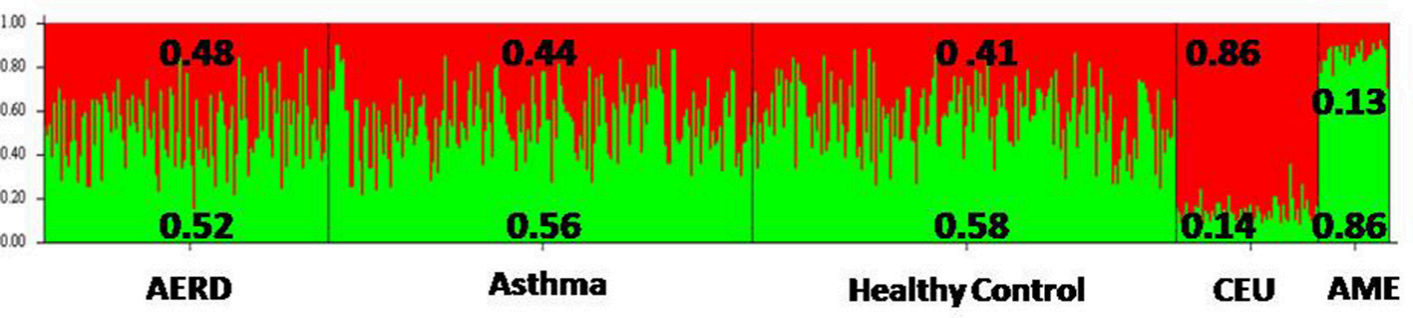
Frequency of ancestry of the three groups of study, according to the two principal ancestral groups that integrate Mexican Mestizo population. AERD, Aspirin-Exacerbated Respiratory Disease; CEU, Caucasian European; AME, Amerindian.

The three groups had a similar proportion of genetic ancestry according to the two principal population groups (CEU, Caucasian and AME, Amerindian) that integrate the Mexican mestizo population. AERD had 52% of AME and 48% of CEU; asthma had 56% AME and 44% CEU; and HC had 41% CEU and 58% AME. The F_ST_ test did not identify any significant difference among the three groups, but there was a difference when the groups were compared with CEU and AME ancestry markers (*p* = 0.005) Figure 3 and Supplementary Table 1.

#### Allele frequency comparison

In AERD vs. HC, we identified 22 associated SNPs, with 11 SNPs associated with risk in 9 genes (*ACE, MS4A2, FSIP, IL10, TBXAS1, FANCC, FCERIG, KIFC3*, and *ANX4*). Two SNPs were strongly associated: *ACE* rs4309 (C allele *p* = 0.0001, OR = 1.92, CI 95% = 1.37–2.69) and *MS4A2* rs573790 (C allele *p* = 0.0002, OR = 1.94, CI 95% = 1.35–2.79). By contrast, 11 SNPs in 5 genes (*PPARG, IL10, RG7SBP, TBXAS1*, and *FANCC*) were associated with protection Table 2 show the statistical analysis.

**Table 2:** Allele frequency comparison between AERD vs. Healthy Control Group.

**Chr**	**Gene**	**SNP**	**Minor allele**	**AF**		***p***	**OR**	**CI 95%**
				**AERD**	**HC**			
17	*ACE*	rs4309	C	0.4832	0.3268	0.0001257	1.926	1.37–2.69
11	*MS4A2*	rs573790	C	0.375	0.236	0.0002506	1.943	1.35–2.77
17	*ACE*	rs4293	G	0.4375	0.2989	0.0005139	1.825	1.29–2.56
15	*FSIP*	rs2631700	T	0.5583	0.4242	0.0013	1.716	1.23–2.38
11	*MS4A2*	rs502581	A	0.2542	0.1517	0.001875	1.906	1.26–2.87
3	*PPARG*	rs2960421	G	0.1167	0.2135	0.002256	0.4866	0.30–0.77
1	*IL10*	rs1554286	T	0.325	0.4438	0.003626	0.6034	0.42–0.84
5	*RGS7BP*	rs6870654	C	0.2417	0.3464	0.006381	0.6014	0.41–0.86
7	*TBXAS1*	rs13239058	T	0.1458	0.2318	0.009583	0.5657	0.36–0.87
3	*PPARG*	rs4135275	G	0.2125	0.3073	0.01044	0.6084	0.41–0.89
1	*IL10*	rs1800872	A	0.3625	0.4605	0.01765	0.6663	0.47–0.93
1	*IL10*	rs1800896	G	0.325	0.2374	0.01841	1.546	1.07–2.22
3	*PPARG*	rs1875796	T	0.425	0.5169	0.02771	0.6909	0.49–0.96
7	*TBXAS1*	rs10487667	G	0.3125	0.3989	0.03161	0.685	0.48–0.96
2	*ANX4*	rs7588022	C	0.3625	0.2809	0.03524	1.456	1.02–2.06
9	*FANCC*	rs1326188	C	0.075	0.1285	0.03807	0.5499	0.31–0.97
15	*FSIP*	rs2631702	G	0.4625	0.3799	0.04431	1.405	1.00–1.95
1	*FCER1G*	rs4489574	T	0.4792	0.3966	0.04571	1.399	1.00–1.94
1	*IL10*	rs3024498	G	0.2292	0.1638	0.04653	1.517	1.00–2.29
1	*FCER1G*	rs7528588	G	0.2125	0.2849	0.04666	0.6772	0.46–0.99
5	*KIFC3*	rs2285700	T	0.4	0.3212	0.04817	1.409	1.00–1.98
7	*TBXAS1*	rs6962291	A	0.3875	0.4689	0.04955	0.7165	0.51–0.99

*AERD, Aspirin-Exacerbated Respiratory Disease; ACE, angiotensin I converting enzyme; AF, Allele frequency; ANX4, annexin A4; CI 95%, Confidence interval at 95%; Chr, Chromosome; FANCC, Fanconi anemia complementation group C; FCER1G, Fc fragment of IgE receptor Ig; FISP, interleukin 24; HC, Healthy Control; IL10, interleukin 10; KIFC3, kinesin family member C3; MS4A2, membrane spanning 4-domains A2; OR, Odds ratio; PPARG, peroxisome proliferator activated receptor gamma; RGS7BP, regulator of G protein signaling 7 binding protein; SNP, single nucleotide polymorphism; TBXAS1, thromboxane A synthase 1*.

In the comparison between the AERD group vs. asthma, we detected 14 SNPs associated with risk in 9 genes (*ACE, FSIP, TBXAS1, IL10, IL1B, CYP2C19, ANX4, TBXAS1*, and *IL13*), and 5 SNPs associated with protection in 4 genes (*IL10, PTGER2, OBSCN*, and *PPARG*). Similar to the previous comparison, *ACE* rs4309 was associated with risk (*p* = 0.0001, OR = 1.92, CI 95% = 1.37–2.69) (Table 3). Regard rs4309 of *ACE*, in the comparison AERD vs. (HC or asthma) the result was statistically significant after Bonferroni correction (factor of 311), *p* = 0.03 in both comparisons.

**Table 3:** Allele frequency comparison between AERD vs. Asthma group.

**Chr**	**Gene**	**SNP**	**Minor allele**	**AF**		***p***	**OR**	**CI 95%**
				**AERD**	**Asthma**			
17	*ACE*	rs4309	C	0.4832	0.3268	0.0001257	1.926	1.37–2.69
17	*ACE*	rs4293	G	0.4375	0.2877	0.0001634	1.926	1.36–2.71
6	*FSIP*	rs2631700	T	0.5583	0.427	0.001642	1.697	1.22–2.36
7	*TBXAS1*	rs2072190	C	0.4708	0.3436	0.001803	1.7	1.21–2.37
7	*IL10*	rs3024498	G	0.2292	0.1313	0.001813	1.967	1.28–3.02
1	*TBXAS1*	rs2269997	C	0.5292	0.4011	0.002096	1.678	1.20–2.33
7	*IL1B*	rs16944	G	0.458	0.3436	0.005023	1.614	1.15–2.25
1	*IL10*	rs1554286	T	0.325	0.4354	0.006754	0.6244	0.44–0.87
1	*IL10*	rs1800896	G	0.325	0.2263	0.007384	1.647	1.14–2.37
7	*CYP2C19*	rs10786172	A	0.4875	0.387	0.01513	1.507	1.08–2.09
10	*ANX4*	rs7588022	C	0.3625	0.2709	0.01742	1.53	1.07–2.17
4	*TBXAS1*	rs757760	A	0.1542	0.09497	0.02827	1.737	1.05–2.85
7	*IL10*	rs1800872	A	0.3625	0.4522	0.02926	0.6887	0.49–0.96
15	*IL13*	rs20541	C	0.4417	0.3567	0.03718	1.426	1.02–1.99
5	*FSIP*	rs2631702	G	0.4625	0.3771	0.03749	1.421	1.02–1.98
15	*FSIP*	rs2411300	A	0.4125	0.3296	0.03879	1.428	1.01–2.00
15	*PTGER2*	rs1409165	C	0.1083	0.1685	0.04022	0.5994	0.36–0.98
1	*OBSCN*	rs4653544	A	0.1208	0.1816	0.0455	0.6195	0.38–0.99
1	*PPARG*	rs2960421	G	0.1167	0.176	0.04778	0.6184	0.38–0.99

*AF, Allele frequency; ACE, angiotensin I converting enzyme; ANX4, annexin A4; CI 95%, Confidence interval at 95%; Chr, Chromosome; CYP2C19, cytochrome P450 family 2 subfamily C member 19; FSIP, interleukin 24, IL10- interleukin 10, IL13- interleukin 13, IL1B- interleukin 1B; OBSCN, obscurin, cytoskeletal calmodulin and titin-interacting; OR, Odds ratio; PPARG, peroxisome proliferator activated receptor gamma; PTGER2, prostaglandin E receptor 2; RhoGEF, TBXAS1, thromboxane A synthase 1*.

**Figure 4:**
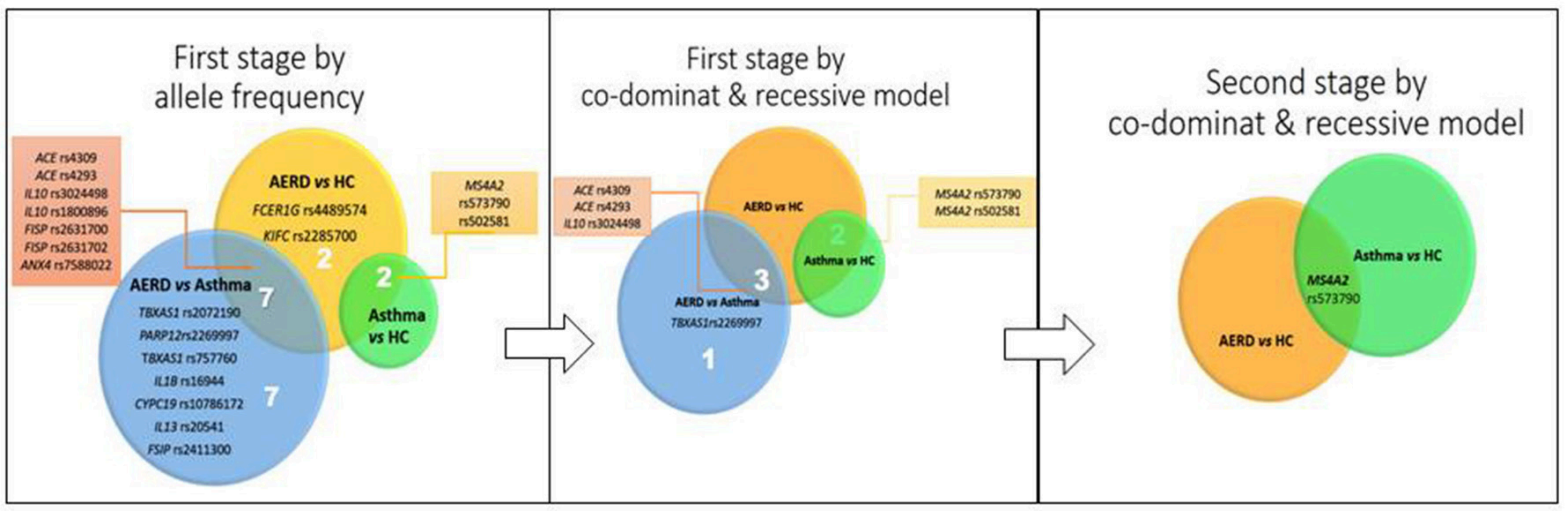
Sequence of result analysis according to stage.

Finally, in the asthma vs. HC comparison, 9 SNPs were identified, with 2 associated with risk in the *MS4A2* gene (rs573790 *p* = 0.001, OR = 1.70, CI 95% = 1.21-2.37), and 7 SNPs associated with protection in 4 genes (*TBXAS1, FANCC, CYSLTR2*, and *PTGER3*) (Table 4). Figure 4 shows the genes and SNPs associated with risk in the three comparisons.

**Table 4:** Allele frequency comparison between Asthma group vs. Healthy control.

**Chr**	**Gene**	**SNP**	**Minor allele**	**AF**		***p***	**OR**	**CI 95%**
				**Asthma**	**HC**			
11	*MS4A2*	rs573790	C	0.3455	0.236	0.001288	1.709	1.23–2.37
7	*TBXAS1*	rs2072190	C	0.3436	0.4581	0.001768	0.6191	0.45–0.83
11	*MS4A2*	rs502581	A	0.2346	0.1517	0.005006	1.715	1.17–2.50
7	*TBXAS1*	rs2269997	C	0.4011	0.5028	0.006428	0.6624	0.49–0.89
9	*FANCC*	rs1326188	C	0.07303	0.1285	0.01387	0.5344	0.32–0.88
7	*TBXAS1*	rs17161326	A	0.2612	0.3455	0.01446	0.6699	0.48–0.92
13	*CYSLTR2*	rs912278	C	0.3268	0.4134	0.01642	0.6889	0.50–0.93
14	*PTGER3*	rs1254600	T	0.3436	0.4302	0.01737	0.6933	0.51–0.93
7	*TBXAS1*	rs13239058	T	0.162	0.2318	0.0188	0.6406	0.44–0.93

*AF, Allele frequency; HC, Healthy Control; CI 95%, Confidence interval at 95%; Cr, Chromosome; CYSLTR2, cysteinyl leukotriene receptor 2; FANCC, Fanconi anemia complementation group C, MS4A2, membrane spanning 4-domains A2; OR, Odds ratio; PTGER3, prostaglandin E receptor 3, SNP, single nucleotide polymorphism; TBXAS1, thromboxane A synthase 1*.

#### Genetic models

All SNPs associated with risk were evaluated using the co-dominant (CM) and recessive model (RM). We show the positive results associated with risk in the three different comparisons.

AERD vs. HC *MS4A2* rs57370 CC genotype (CM) had a significant association *p* = 0.001, OR = 7.8 CI 95% = 2.45–25.28 and *p* = 0.001, OR = 5.7, CI 95% = 1.85–18.01 (RM). *ACE* rs4309 CC gave *p* = 0.002, OR = 3.32, CI 95% = 1.65–6.68 (CM) and *p* = 0.02, OR = 2.07 CI 95% = 1.11–3.84 (RM). Finally, *ACE* rs4293 GG was *p* = 0.02, OR = 3.4, CI 95% = 1.61–7.37 (CM) and *p* = 0.01, OR = 2.45, CI 95% = 1.21–4.95 (RM) (Table 5).

**Table 5:** Co-dominant and recessive genetic models of principals SNP associated to risk of AERD.

**Gene/SNP**	**AERD**	**HC**	**Asthma**	**AERD vs. HC**		**AERD vs. Asthma**		**Asthma vs. HC**	
	***n* = 120 (%)**	***n* = 179 (%)**	***n* = 179 (%)**	***p***	**OR (CI95%)**	***p***	**OR (CI95%)**	***p***	**OR (CI95%)**
***ACE*** **rs4309**									
TT	32 (26.66)	84 (46.92)	77 (43)	0.0002	1	0.0001	1	0.24	1
TC	61 (50.83)	73 (40.78)	87 (48.60)		2.26 (1.32–3.86)		1.68 (0.99–2.85)		1.3 (0.83–2.01)
CC	27 (22.5)	22 (12.29)	15 (8.37)		3.32 (1.65–6.68)		4.33 (2.03–9.20)		0.74 (0.36–1.53)
TT+TC	93 (77.5)	157 (87.71)	164 (91.63)	0.02	2.07 (1.11–3.84)	0.001	3.17 (1.6–6.26)	0.29	1.53 (0.76–3.06)
CC	27 (22.5)	22 (12.29)	15 (8.37)						
***ACE*** **rs4293**									
AA	37 (30.83)	87 (48.60)	85 (47.48)	0.02	1	0.0001	1		1
AG	61 (50.83)	77 (43.01)	85 (47.48)		1.8 (1.11–3.14)		1.64 (0.99–2.73)	0.38	1.12 (0.73–1.73)
GG	22 (18.33)	15 (8.37)	9 (5.02)		3.4 (1.61–7.37)		5.61 (2.36–13.35)		0.61 (0.25–1.47)
AA+GG	98 (81.67)	164 (91.63)	170 (94.98)	0.01	2.45 (1.21–4.95)	0.0004	4.2 (1.87–9.57)	0.29	1.72 (0.73–4.05)
GG	22 (18.33)	15 (8.37)	9 (5.02)						
***MS4A2*** **rs573790**									
TT	44 (36.66)	99 (55.33)	80 (44.69)	0.0001	1	0.24	1	0.001	1
TC	62 (51.66)	76 (42.45)	75 (41.89)		1.8 (1.12–2.99)		1.5 (0.91–2.47)		1.22 (0.79–1.88)
CC	14 (11.66)	4 (22.34)	24 (13.4)		7.8 (2.45–25.28)		1.06 (0.49–2.25)		7.42 (2.47–22.27)
TT+TC	106 (88.34)	175 (77.66)	155 (86.60)	0.001	5.7 (1.85–18.01)	0.79	0.85 (0.42–1.72)	0.0001	6.77 (2.29–19.95)
CC	14 (11.66)	4 (22.34)	24 (13.4)						
***MS4A2*** **rs502581**									
CC	64 (53.33)	128 (71.5)	107 (59.77)	0.004	1	0.23	1	0.01	1
AC	51 (42.50)	48 (26.81)	60 (33.51)		2.12 (1.29–3.48)		1.42 (0.87–2.30)		1.49 (0.94–2.36)
AA	5 (4.16)	3 (1.67)	12 (6.70)		3.33 (0.77–14.38)		0.69 (0.23–2.06)		4.78 (1.31–17.39)
CC+AC	115 (95.84)	176 (98.83)	167 (93.30)	0.34	2.5 (0.59–10.87)	0.5	1.65 (0.56–4.81)	0.03	4.21(1.16–15.2)
AA	5 (4.16)	3 (1.67)	12 (6.70)						
**IL10 rs3024498**									
AA	74 (61.66)	123 (68.71)	135 (75.41)	0.01	1	0.003	1	0.28	1
AG	37 (30.83)	54 (30.16)	41 (22.9)		1.13 (0.68–1.89)		1.63 (0.96–2.76)		0.69 (0.43–1.11)
GG	9 (7.5)	2 (1.11)	3 (1.67)		7.47 (1.57–35.56)		5.43 (1.42–20.68)		1.36 (0.22–8.31)
AA+AG	111 (92.5)	177 (98.89)	176 (98.33)	0.02	7.17 (1.52–33.82)	0.002	4.75 (1.26–17.97)	1	0.66 (0.10–4.01)
GG	9 (7.5)	2 (1.11)	3 (1.67)						
***TBXAS1 rs2269997***									
TT	29 (24.16)	50 (27.93)	69 (38.54)	0.7	1	0.002	1	0.02	1
TC	55 (45.83)	78 (43.57)	78 (45.37)		1.24 (0.70–2.19)		1.67 (0.96–2.92)		0.73 (0.45–1.19)
CC	36 (30)	51 (28.49)	32 (17.87)		1.26 (0.67–2.36)		2.67 (1.40–5.09)		0.47 (0.26–0.83)
TT+TC	84 (69.99)	128 (71.51)	147 (82.13)	0.87	0.92 (0.55–1.54)	0.02	1.96 (1.13–3.41)	0.02	0.54(0.33–0.90)
CC	36 (30)	51 (28.49)	32 (17.87)						

*AERD, Aspirin-Exacerbated Respiratory Disease; ACE, angiotensin I converting enzyme; CI 95%, Confidence interval at 95%; HC, Healthy Control; IL10, interleukin 10; MS4A2, membrane spanning 4-domains A2; OR, Odds ratio; SNP, single nucleotide polymorphism; TBXAS1, thromboxane A synthase 1*.

In AERD vs. asthma, we detected 4 SNPs associated in three genes: *TBXAS1* rs2269997 CC *p* = 0.002, OR = 2.67, CI 95% = 1.40–5.0 (CM), and *p* = 0.02, OR = 1.96, CI 95% = 1.13–3.34 (RM). For *ACE*, two SNP are associated: rs4309 CC *p* = 0.0001, OR = 4.33, CI 95% = 2.03–9.20 and *p* = 0.001, OR = 3.17, CI 95% = 1.6–6.26 (CM and RM, respectively); and rs4293 GG *p* = 0.0001, OR = 5.61, CI 95% = 2.36–13.35 and *p* = 0.0004, OR = 4.20, CI 95% = 1.87–9.57. *IL10* rs3024498 GG gave *p* = 0.003, OR = 5.43, CI 95% = 1.42–20.98 (CM) and *p* = 0.002, OR = 4.75 CI 95% = 1.26–17.95 (RM) (Table 5).

For asthma vs. HC comparison, *MS4A2* rs573790 CC gave *p* = 0.001, OR = 7.42, CI 95% = 2.47–22.27 (CM) and *p* = 0.001 OR = 6.77 CI 95% = 2.29-19.95 (RM). *MS4A2* rs502581 AA gave *p* = 0.01, OR 4.78, CI 95% 1.31–17.39 (CM) and *p* = 0.03 OR = 4.21, CI 95% 1.16–15.20 (RM) (Table 5 and Figure 4).

### Stage 2

#### Demographic and clinical data

In the second stage, 343 subjects were included: 104 patients with AERD, 107 with asthma, and 132 healthy controls. Demographic data were similar to the first stage. The HC group was younger than the AERD and asthma group (*p* < 0.05), and the prevalence of the female gender was approximately 70% in the three groups. Counts of serum eosinophils were greater in AERD patients vs. asthma and HC groups (*p* = 0.03, *p* = 0.001). Total IgE levels were higher in the asthma group compared with AERD and controls (*p* = 0.001), and allergic sensitivity was a principal characteristic in asthma patients vs. the other two groups (*p* < 0.01). For the lung function test, the reversibility was statistically significative in the asthma group only (Table 1).

#### Allelic and genetic models

In the second stage, we investigated only those SNPs associated with risk in the first stage. For the SNP rs573790 (*MS4A2)*, the minor allele is C, and it had a proportion of 33.17% in AERD, 32.24% in asthma and 32.57% in healthy controls (*p* > 0.05). In the co-dominant model, we found a statistical significance when evaluating the CC genotype vs. (TT or CT) in AERD vs. HC *p* = 0.008, OR = 2.27, and this was similar in the asthma group vs. HC *p* = 0.03, OR = 1.93 (Figure 4). Statistical significance as well as its risk association are maintained only in the comparison of AERD vs. HC in the recessive model [CC vs. (TT+CT)], *p* = 0.03 OR = 3, but not in asthma patients vs. controls (*p* = 0.09). For *TBXAS1* rs757760, *MS4A2* rs502581, *IL10* rs3024498, and *ACE* (rs4293 and rs4309), we did not detect any statistical association comparing AERD vs. asthma/HC or asthma vs. HC, in the allelic, recessive and codominant models (*p* > 0.05) (Table 6).

**Table 6:** Co-dominant and recessive genetics models of Stage 2.

**Gene/SNP**	**AERD**	**HC**	**Asthma**	**AERD vs. HC**		**AERD vs. Asthma**		**Asthma vs. HC**	
	***n* = 104 (%)**	***n* = 132 (%)**	***n* = 107 (%)**	***p***	**OR (CI 95%)**	***p***	**OR (CI 95%)**	***p***	**OR (CI 95%)**
***ACE*** **rs4309**									
TT	40 (38.46)	44 (37.33)	40 (37.23)	0.65	1	0.58	1	0.25	1
CT	50 (48.35)	66 (50.00)	57 (53.17)		0.83 (0.47–1.46)		0.87 (0.49–1.56)		0.95 (0.54–1.65)
CC	14 (13.18)	22 (16.66)	10 (9.57)		0.70 (0.31–1.55)		1.40 (0.55–3.52)		0.50 (0.21–1.18)
TT+CT	90 (86.54)	110 (83.33)	97 (90.67)	0.61	0.77 (0.37–1.60)	0.46	1.50 (0.63–3.56)	0.14	0.51 (0.23–1.14)
CC	14 (13.46)	22 (16.67)	10 (9.35)						
T	130 (62.50)	154 (58.33)	137 (64.02)	0.41	0.84 (0.57–1.21)	0.82	1.06 (1.71–1.58)	0.24	0.78 (0.54–1.14)
**C**	78 (37.50)	110 (41.67)	77 (35.92)						
***ACE*** **rs4293**									
AA	51 (49.03)	64 (48.48)	49 (45.79)	0.94	1	0.31	1	0.44	1
AG	40 (38.46)	53 (40.15)	50 (46.72)		0.94 (0.54–1.64)		0.76 (0.43–1.36)		1.23 (0.72–2.10)
GG	13 (12.5)	15 (11.36)	8 (7.47)		1.08 (0.47–2.49)		1.56 (0.59–4.09)		0.69 (0.27–1.77)
AA+AG	91 (87.5)	117 (88.63)	99 (92.52)	0.94	1.11 (0.50–2.45)	0.32	1.76 (0.70–4.46)	0.42	0.63 (0.25–1.54)
GG	13 (12.5)	15 (11.36)	8 (7.47)						
A	142 (68.26)	181 (68.56)	148 (69.15)	1	1.01 (0.68–1.49)	0.92	1 (0.69–1.57)	0.96	0.97 (0.65–1.43)
G	66 (31.73)	83 (31.43)	66 (30.84)						
***MS4A2*** **rs573790**									
TT	50 (48.07)	53 (40.15)	51 (47.66)	0.008	1	0.85	1	0.03	1
CT	39 (37.5)	72 (54.54)	43 (40.18)		0.57 (0.33–0.99)		0.92 (0.51–1.65)		0.62 (0.36–1.06)
CC	15 (14.42)	7 (5.33)	13 (12.14)		2.27 (0.85–6.03)		1.17 (0.50–2.72)		1.93 (0.71–5.22)
CT+TT	89 (85.57)	125 (94.69)	94 (87.85)	0.03	3 (1.17–7.68)	0.77	1.21 (0.54–2.70)	0.09	2.46 (0.94–6.43)
CC	15 (14.42)	7 (5.30)	13 (12.14)						
T	139 (66.82)	178 (67.42)	145 (67.75)	0.96	1.02 (0.69–1.51)	0.92	1.04(0.69–1.56)	1	0.98 (0.67–1.44)
C	69 (33.17)	86 (32.57)	69 (32.24)						
***MS4A2*** **rs502581**									
GG	61 (58.65)	84 (63.63)	73 (68.22)	0.33	1	0.34	1	0.31	1
GT	36 (34.61)	35 (26.51)	29 (27.10)		1.41 (0.80–2.50)		1.48 (0.81–2.69)		0.95 (0.53–1.70)
TT	7 (6.73)	13 (9.84)	5 (4.67)		0.74 (0.27–1.96)		1.67 (0.50–5.54)		0.44 (0.15–1.30)
GG+GT	97 (93.27)	119 (90.15)	102 (95.32)	0.53	0.66 (0.25–1.72)	0.72	1.47 (0.45–4.79)	0.2	0.44 (0.15–1.30)
TT	7 (6.73)	13 (9.84)	5 (4.67)						
G	158(75.96)	203(76.89)	175(81.77)	0.89	1.05 (0.68–1.61)	0.17	1.42(0.88–2.27)	0.23	0.74 (0.47–1.16)
T	50(24.03)	61(23.10)	39(18.22)						
***IL10*** **rs3024498**									
TT	75 (72.11)	89 (67.42)	77 (71.96)	0.43	1	0.52	1	0.69	1
CT	23 (22.11)	38 (28.78)	27 (13.77)		0.71 (0.22–0.74)		0.87 (0.46–1.65)		0.82 (0.45–1.46)
CC	6 (5.76)	5 (3.78)	3 (2.80)		1.42 (0.09–0.67)		2.05 (0.49–8.51)		0.69 (0.16–2.99)
TT+CT	98 (94.23)	127 (96.22)	104 (97.19)	0.54	1.5 (0.46–5.24)	0.46	2.12 (0.51–8.72)	0.73	0.73 (0.17–3.13)
CC	6 (5.76)	5 (3.78)	3 (2.80)						
T	173(83.17)	216(81.81)	181(84.57)	0.71	0.91 (0.56–1.47)	0.79	1.1(0.66–1.86)	0.49	0.82 (0.50–1.33)
C	35(16.82)	48(18.18)	33(15.42)						
***TBXAS1*** **rs757760**									
GG	26 (25)	40 (30.30)	34 (31.77)	0.36	1	0.34	1	0.96	1
AG	57 (54.80)	60 (45.45)	48 (44.85)		1.46 (0.79–2.69)		1.55 (0.81–2.94)		0.94 (0.51–1.70)
AA	21 (20.19)	32 (24.24)	25 (23.36)		1.0 (0.48–2.11)		1.09 (0.50–2.37)		0.91 (0.45–1.84)
GG+AG	83 (79.80)	100 (75.76)	82 (76.63)	0.55	0.79 (0.42–1.47)	0.69	0.82 (0.43–1.59)	0.99	0.95 (0.52–1.73)
AA	21 (20.19)	32 (24.24)	25 (23.36)						
G	109 (52.4)	140 (53.03)	116 (54.2)	0.96	1.02 (0.71–1.47)	0.74	1.07 (0.73–1.57)	0.86	0.95 (0.66–1.36)
A	99 (47.59)	124 (46.96)	98 (45.79)						

*AERD, Aspirin Exacerbated Respiratory Disease; ACE, angiotensin I converting enzyme; CI 95%, Confidence interval at 95%; HC, Healthy Control; IL10, interleukin 10; MS4A2, membrane spanning 4-domains A2; OR, Odd ratio; SNP, single nucleotide polymorphism; TBXAS1, thromboxane A synthase 1*.

There was no relationship/association with the splicing process or microRNA generation based on the respective analysis software.

## Discussion

In the present study, we analyzed 53 candidate genomic regions, spanning in 19 chromosomes, associated with AERD previously in the literature between 1997 and 2014, using a tag SNP strategy in Mexican mestizo patients with this disease. The research was developed in two stages. First, we identified six SNPs in four genes by GoldenGate analysis, followed by qPCR validation in other independent groups. *MS4A2* rs573790 was the only SNP that supports the association with the risk in the two stages.

AERD is considered a particular phenotype of asthma and chronic rhinosinusitis (CRS). It occurs in 15% of patients with severe asthma (Kennedy et al., 2016) and 16% of those with CRS (Stevens et al., 2017). This low prevalence could result in under diagnosed patients with AERD worldwide. All our patients met the three characteristics of AERD, not only asthma with NSAID intolerance. We had a predominance of female gender, similar to Europeans (69%) at the age of 40 years on average (Szczeklik et al., 2000; Bavbek et al., 2012).

The allergy sensitivity was approximately 50% in AERD patients. Other reports state that this characteristic can be as high as 85% (Stevens et al., 2017). Most patients had an acceptable lung function similar to other recent findings (Bochenek et al., 2015). To demonstrate aspirin hypersensitivity in the first stage, we included patients with a positive challenge. Because 85% of patients with the antecedent of lung reaction have a positive challenge (Nizankowska-Mogilnicka et al., 2007), we only enrolled patients who had a reaction to NSAID or ASA who were treated in an emergency room in the last 12 months.

Using new methods, such as GWAS technology, new candidate genes in AERD were identified, such as HLA-DPB1 (Park et al., 2013). There are few studies of candidate genes in AERD, and most were detected in Asian populations. However, some findings are conflicting, and the majority of reported associations lack of replication (Dahlin and Weiss, 2016). One study in a different population was conducted among Spanish people. They re-analyzed genes in the AA pathway, and a new SNP of *ALOX15* and *PTGS-1* were found to be associated with AERD risk in comparison with asthma and healthy subjects (Ayuso et al., 2015).

The need to validate results found in other populations for use as genetic markers capable of predicting this disease led to multistage studies in a population with different genetic backgrounds (Boezen, 2009). Thus, we selected all genes previously reported to be associated with AERD (*n* = 384 SNPs) and assessed their genotypes in an Illumina 384 SNP custom GoldenGate array. The SNP rs573790 in the *MS4A2* gene was at the top of variants associated with AERD in both stages of our study. MS4A is a large family gene, clustered in chromosome 11q12. *MS4A2* encodes the β subunit of high-affinity immunoglobulin E receptor (FcεRIβ), considered a maturation marker for eosinophils and MC. Studies reported that the *MS4A2* gene is expressed as multiple splice variants that are predicted to encode different protein isoforms, and some polymorphisms (I181L, V183L, and E237G) were associated with atopy and other diseases (Ma et al., 2015; Eon Kuek et al., 2016).

Eosinophils and MCs play a role in the pathogenesis of AERD. Their mediators, eosinophil cationic protein and major basic protein, are linked to the exacerbation and pathogenesis of AERD (Rodríguez-Jiménez et al., 2018). Other studies reported that activated MCs are higher in CRS with NP in AERD compared with those from ATA (Varga et al., 1999) and contribute to the production of leukotrienes (Choi et al., 2013).

The roll of *MS4A2* in AERD is not fully understood. Its protein, FcεRIβ, is an essential component of the heterotetramer that comprises the IgE receptor FcεRIα, FcεRIβ and FcεRIγ (αβγ2) in eosinophils and MC (Potaczek and Kabesch, 2012). It participated in intracellular signaling, amplifying FcεRIγ-mediated signaling. In mice, FcεRIβ also amplifies FcεRI signaling by promoting the assembly, stabilization, and trafficking of the receptor complex to the cell surface (Cruse et al., 2013).

In terms of genetic epidemiology, a meta-analysis of *MS4A2* polymorphisms and its association with asthma in Asian subjects did not find any association of E237G with the disease or its atopic phenotype; however, −109C/T in asthma has a significantly decreased risk of disease based on the allele (C vs. T), whereas there is no evidence of association in genetics models (Yao et al., 2015). Kim and coworkers found that the −109T>C polymorphisms (TT vs. TC+CC) are associated with risk in patients with AERD (with *Staphylococcus* B enterotoxin) vs. ATA and the control group in a Korean population (Kim et al., 2006).

*MS4A2* was evaluated in Latino asthma patients (Puerto Ricans and Mexicans from native country and residents in the USA) as part of replicative genetic study on asthma. Galanter and collaborators showed that this gene was associated with asthma in Mexicans, but not in Puerto Rican patients (Galanter et al., 2011).

The SNP rs573790 is localized in the 5′UTR region of the *MS4A2* gene. This type of polymorphism is localized in a non-coding region, usually related to alteration in the function or structure of RNA (Sadee, 2009); however, in our study, we did not detect this using software tools. The C allele (minor allele) is increased in AERD cases, and this frequency reaches 43% in Mexican residents in Los Angeles, USA (Auton et al., 2015). Interestingly, in a Mexican population, this frequency is lower than 32% in controls. In addition, our data showed that this SNP deviates from Hardy-Weinberg equilibrium, which may be due to the young genetic structure (mixture among Caucasian and Amerindian) of Mexican mestizo population (Pérez-Rubio et al., 2016). This is the first time that rs573790 (CC genotype) is associated with a human disease of any type (Zerbino et al., 2017).

The angiotensin-converting enzyme (ACE), a key enzyme of the renin angiotensin system, is mainly expressed in the lung and plays an important role in the pathogenesis of asthma (Lee et al., 2000). Its function consists of inactivating a wide range of inflammatory peptides as kinins and substance P (Christiansen et al., 1987). Polymorphisms in the *ACE* gene were implicated in risk of asthma and AERD (Kim et al., 2008; Liu et al., 2013). The SNP rs4309 in *ACE* was associated with risk in the first stage in our study, but not in the second. An analysis of surrounding regions shows that this synonymous polymorphism (C) is within the rich zone of CpG islands (Li and Dahiya, 2002). This type of DNA region is strong, resistant to denaturation and is difficult to hybridize primers in conventional qPCR; therefore, it may not be the ideal technique for validating this finding (Flores-Juárez et al., 2016).

Genetic studies in AERD have explored genes, single nucleotide polymorphisms, variable number tandem repeats, HLA alleles and exomes, using diverse techniques, however most of them were developed in Asian and Caucasian populations. It is necessary to validate their positives results in a second population, particularly in those with different genetic backgrounds, to strengthen the role of genetic susceptibility in AERD physiopathology, and to provide a framework for personalized medicine. Our current research presents by first time a replicative two-stage genetic association study in AERD, in a population including Amerindian and Caucasian ancestral contribution. We think that this approach strengthen our main findings.

In our study, rs573790 in *MS4A2* was the only polymorphism associated with AERD risk. Additional studies spanning *MS4A2* gene region, employing sequencing techniques, could help to identify other SNPs related to AERD pathogenesis.

## Author contributions

GFP-R enrolled patients, review of literature, DNA isolation, development of molecular biology techniques, bioinformatics' analysis, manuscript redaction. GP-R bioinformatics' and statistical analysis. EA-O development of molecular biology techniques, bioinformatics' analysis. FR-J, EB-O, NA-F, KEX-R, EH-J, and BAF-G enrolled patients. AEC enrolled patients, development of molecular biology techniques. LMT development of molecular biology techniques, manuscript redaction. RF-V development of molecular biology techniques, bioinformatics' analysis, manuscript redaction.

### Conflict of interest statement

The authors declare that the research was conducted in the absence of any commercial or financial relationships that could be construed as a potential conflict of interest.
