# Supplementary material for: MS4A2-rs573790 Is Associated With Aspirin-Exacerbated Respiratory Disease: Replicative Study Using a Candidate Gene Strategy
Source: Front Genet. 2018 Sep 11;9:363. doi: 10.3389/fgene.2018.00363 (PMC6141666; doi:10.3389/fgene.2018.00363)
Supplement: Supplementary file 1 [file Table_1.DOCX]

**Table supplementary 1. Ancestral contribution of each group (cross table)**

| Group | Ancestral contribution * | | Asthma | AERD | CEU ^ǂ^ | AME (native)^ǂ^ |
| --- | --- | --- | --- | --- | --- | --- |
|  | CEU | AME (native) |  |  |  |  |
| Healthy control | 0.41 | 0.58 | 0.59 | 0.23 | 0.005 | 0.005 |
| Asthma | 0.44 | 0.56 |  | 0.47 | 0.005 | 0.005 |
| AERD | 0.48 | 0.52 |  |  | 0.005 | 0.005 |

*Ancestral contribution (AC) of each study group with respect to the two reference population that integrated the ancestry of Mexican-mestizo. ǂ Reference population, AERD- Aspirin-Exacerbated Respiratory Disease, AME-Amerindian, CEU-Caucasian European of Utah. Light shading shows *p* values among the study groups and dark shading shows *p* values among the AC study groups *vs.* reference population.
